# Lessons from the past: resurrecting an ancestral strigolactone receptor

**DOI:** 10.1111/tpj.71189

**Published:** 2026-10-29

**Authors:** Brian C. Mooney

Plants use an array of phytohormones to coordinate their growth and communicate with the organisms around them. Strigolactones (SLs) are a class of butenolide phytohormones with a wide range of physiological roles. First identified as root exudates that could stimulate the germination of parasitic *Striga* plants, they were later found to promote symbioses with arbuscular mycorrhizal fungi and root bacteria. Within the plant, SLs regulate several aspects of development, most notably by suppressing shoot branching (Kelly et al., 2023).

In seed plants, SLs are perceived by DWARF14 (D14), which acts as both a receptor and an α/β‐hydrolase enzyme. D14 binds SLs within a hydrophobic pocket and hydrolyses them using a catalytic triad of serine, aspartate and histidine residues. Ligand perception also triggers a conformational change that enables D14 to interact with the F‐box protein MORE AXILLARY GROWTH 2 (MAX2), leading to the degradation of SUPPRESSOR OF MAX2‐LIKE/DWARF 53 (SMXL/D53) transcriptional repressors and induction of SL‐responsive genes. Intriguingly, however, the relationship between the enzymatic and signalling functions of D14 remains unresolved. Mutating its catalytic aspartate abolishes SL hydrolysis without preventing signalling in *Arabidopsis*, suggesting that ligand binding, rather than hydrolysis, is sufficient to activate the receptor (Seto et al., 2019).

D14 belongs to a larger family of related α/β‐hydrolases that includes KARRIKIN INSENSITIVE 2 (KAI2). KAI2 is a receptor for karrikins, plant growth regulators that structurally resemble SLs and are found in the smoke from burning plants. KAI2 homologues are found throughout land plants and algae, whereas D14 is restricted to seed plants, suggesting that D14 evolved following duplication and neofunctionalisation of an ancestral KAI2‐like protein. Although modern KAI2 and D14 proteins share structural and mechanistic similarities, they have distinct ligand preferences and cannot substitute for one another in *Arabidopsis*. How and when D14 acquired the characteristics of a specialised SL receptor thus remains an intriguing evolutionary question.

Associate Professor Mark Waters at The University of Western Australia first became interested in SL perception through his group's work on karrikins. In particular, the group was curious about the evolutionary changes that led to the distinct activities of KAI2 and D14. Waters wondered whether an early D14 present at the origin of seed plants might retain KAI2‐like characteristics, potentially revealing an intermediate stage in the transition from ancestor to a modern SL receptor. In their new study, Waters and colleagues travelled back along this evolutionary trajectory using ancestral sequence reconstruction (ASR) (Tuckey et al., 2026). ASR uses phylogenetic relationships between modern proteins to infer sequences that may have existed in their common ancestors. Working with collaborators at the University of Leeds, the team reconstructed ancD14, a protein representing the ancestor of D14 orthologues from angiosperms and gymnosperms (clade eu‐D14) as well as a minor sister taxon unique to conifers (termed DLK4) (Figure 1A).

**Figure 1:**
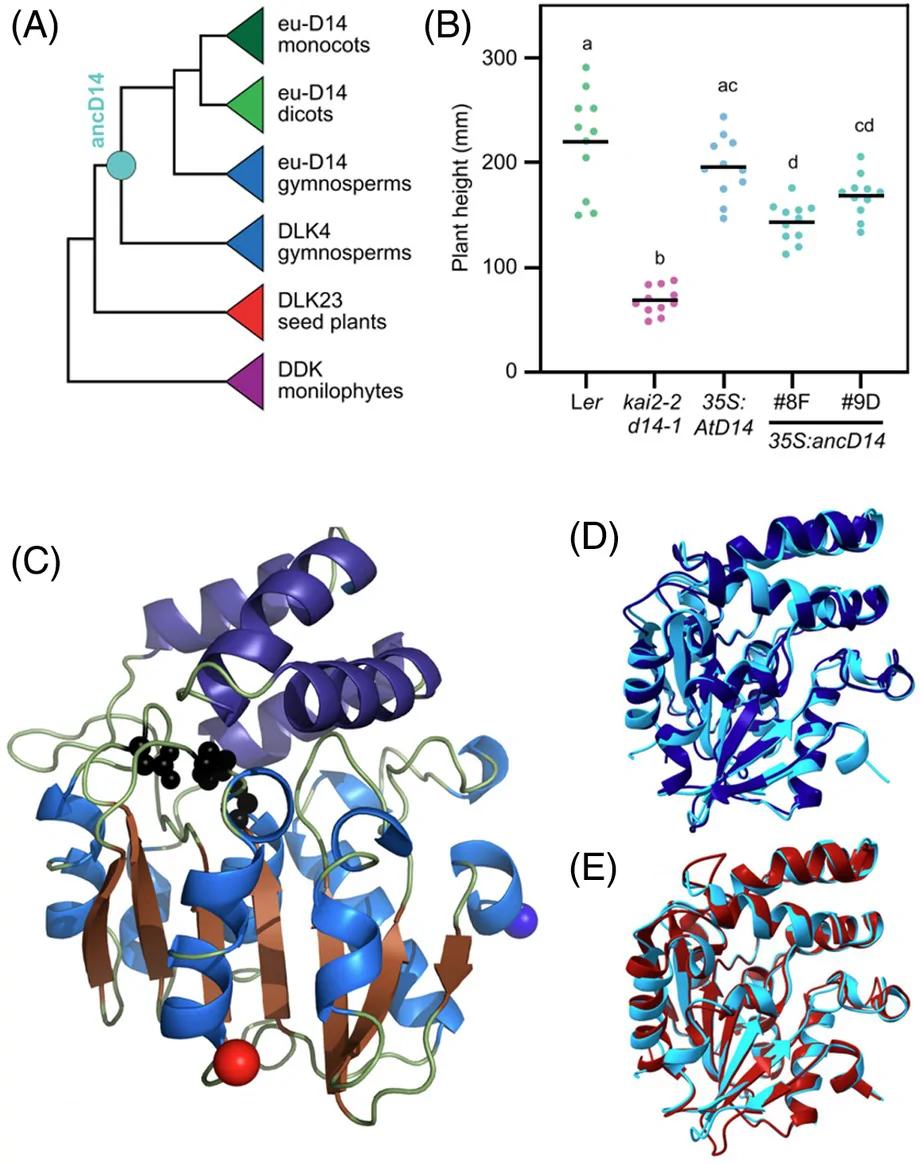
Structure and function of ancD14. (A) AncD14 was designed to represent the ancestor of eu‐D14 and DLK4 sequences from seed plants. Simplified cladogram based on maximum likelihood phylogeny. (B) Plant height measured 18 days after flowering in *kai2 d14* mutants complemented with *AtD14* and *ancD14*. (C) Cartoon representation of ancD14 coloured by secondary structure. The catalytic triad is represented by black balls‐and‐sticks. N and C termini are represented as blue and red spheres, respectively. (D) Structural alignment of ancD14 and AtD14 (Protein Data Bank entry: 4IH4); root mean square deviation of atomic positions (RMSD) = 0.896 Å. (E) Structural alignment of ancD14 and AtKAI2 (Protein Data Bank entry: 4HRX); RMSD = 0.727 Å. Figure adapted from Tuckey et al. (2026).

To test the activity of the ancestral protein, Tuckey et al. (2026) expressed ancD14 in an *Arabidopsis kai2 d14* double mutant. AncD14 partially rescued phenotypes caused by the absence of D14, including increased shoot branching and reduced plant height (Figure 1B). Contrastingly, a delayed flowering phenotype attributed to the *kai2* mutation was not rescued by ancD14. Similarly, ancD14 restored the response to the synthetic SL analogue rac‐GR24, but not to the desmethyl compound rac‐dGR24 preferred by KAI2. Rather than exhibiting the anticipated mixture of KAI2‐ and D14‐like properties, the ancestral protein instead behaved more like the *bona fide* SL receptor.

The authors next investigated whether this functional conservation extended to the biochemical properties of ancD14 using Yoshimulactone Green (YLG), a substrate that closely mimics natural SLs. Like *Arabidopsis thaliana* D14 (AtD14), ancD14 hydrolysed both YLG and its desmethyl derivative dYLG. There were nevertheless several important differences: while ancD14 showed a maximum catalytic activity approximately twice that of AtD14, it had a considerably lower affinity for YLG. Thus, while ancD14 retained the fundamental ligand specificity of the modern D14 receptor, some aspects of its substrate recognition appear to have been refined over time.

To characterise the structural basis for this activity, Tuckey et al. (2026) determined the crystal structure of ancD14 at 1.95 Å resolution. While the overall architecture closely resembled modern D14 (Figure 1C,D), the ancD14 active site contained some unexpected features. The catalytic serine and histidine were positioned closer together, and the orientation of the catalytic serine was more characteristic of KAI2 (Figure 1E). These findings suggest that SL perception can tolerate considerable variation in the configuration of the D14 active site and further indicate that interactions with downstream signalling partners, rather than a rigid ligand‐binding geometry, may have been of primary importance during the functional specialisation of D14.

The authors next used ancD14 to address the relationship between SL hydrolysis and signalling. The catalytic aspartate of ancD14 was replaced with alanine to generate the catalytically inactive ancD14^D216A^. Although this variant could no longer hydrolyse its substrate, it remained as effective as unmodified ancD14 at rescuing the leaf, height and branching phenotypes of *d14* mutants. This result suggests that the separation of SL signalling from ligand hydrolysis is an evolutionarily conserved feature of seed‐plant D14 proteins.

The ancestral receptor also delivered unexpected practical advantages. AncD14 exhibited increased thermostability and more than twice the recombinant protein yield compared with *Arabidopsis* D14. Waters believes that these properties mean that ancD14 could be deployed as a robust biosensor for SLs. The group is already extending the ASR approach to KAI2, where ancestral variants are being developed as molecular probes for karrikin‐like compounds in plants.

Together, these findings suggest that the core identity of D14 as an SL receptor was already established around the emergence of seed plants. Despite altered enzyme kinetics and unusual active‐site geometry, ancD14 already featured the ligand specificity, signalling activity and mechanistic characteristics of its modern descendants. At the same time, its exceptional stability and tractability demonstrate that looking backwards through protein evolution can reveal new paths forward for biological discovery and protein engineering.

## Data Availability

Data sharing not applicable to this article as no datasets were generated or analysed during the current study.
